# Innovative and ecological: integrating ecological momentary assessment into environmental science research

**DOI:** 10.3389/fpsyg.2025.1557055

**Published:** 2025-04-15

**Authors:** Monika Lohani, Ginger Blodgett

**Affiliations:** Applied Cognitive Regulation Laboratory, Department of Psychology, University of Utah, Salt Lake City, UT, United States

**Keywords:** ecological momentary assessment, interdisciplinary research, innovation, ecological advancement, climate change

## Abstract

Ecological momentary assessment (EMA) is a widely used methodology in psychological sciences; however, more broadly, environmental scientists have yet to fully capitalize on the benefits this method offers for gaining a critical understanding of subjective and behavioral responses to environmental factors. EMA enables the collection of experiences and actions occurring in one's natural environment as they unfold over time, allowing researchers to gain contextually informed, dynamic, and longitudinal insights. EMA can provide an accurate understanding of experiences and behaviors relevant to environmental science. To share this perspective, first, we describe current limitations in environmental research that could be addressed through the integration of EMA. Second, we discuss several benefits of adopting EMA in environmental sciences. Finally, we highlight the challenges and considerations involved in integrating EMA. The overarching implication of this work is to foster the interdisciplinary potential and promise of EMA methodology in advancing environmental science research.

## 1 Introduction

*Ecological Momentary Assessment* (EMA, also called experience sampling) is a methodology that enables the collection of experiences and behaviors occurring in one's naturalistic environment in near-real-time (e.g., Stone et al., 2023a; Shiffman et al., 2008; Wilhelm et al., 2006). EMA is a widely used methodology in psychological sciences; however, environmental scientists have yet to maximize the benefits this method offers to gain a comprehensive understanding of experiences and behaviors in naturally occurring contexts. At the same time, affective science has extensively adopted EMA to explore contextual factors and their links to health and wellbeing, and we join recent calls that have highlighted the importance of adopting innovative and varied approaches to inform climate science (Brosch, 2021; Brosch and Sauter, 2023; Clayton and Ogunbode, 2023). Extending these previous suggestions, in the current paper, we highlight that EMA can make important methodological contributions in learning more about dynamic processes relevant to environmental science research.

### 1.1 Goals and target contribution

The goal of the current perspective is to emphasize the benefits of adopting EMA in environmental science research. Accordingly, we first describe the limitations present in environmental science research that can be addressed by EMA methodology. Second, after introducing the EMA methodology, we highlight its relevance and benefits for environmental science research. Finally, we discuss some challenges and considerations when implementing the EMA methodology. The novel contribution of the current work is to showcase the potential and promise of adopting EMA in interdisciplinary environmental science research. While a few studies are beginning to adopt EMA, to our knowledge, very little has been done to clearly introduce and extend this helpful methodology to environmental science research. To address this gap in knowledge, we consider how EMA can be integrated to advance environmental science research. We specifically advocate for its untapped potential to better understand underlying psychological processes in environmental science. We utilize climate change as an example to illustrate this perspective; however, the methodology is applicable to environmental science in general.

### 1.2 Challenges in capturing environment-relevant information in real-time

A large body of literature has captured the significant impact that climate change can have on the economy, health, and society (Gasper et al., 2011; Tol, 2018; McMichael and Lindgren, 2011). Similarly, it has been reliably documented that the feelings, beliefs, and actions of individuals are crucial for understanding and facilitating collective action (Brosch, 2021; Fritsche and Masson, 2021; Klöckner, 2013). Researchers need to rely heavily and frequently on self-reports to explore personal experiences specific to climate change (e.g., Bateman and O'Connor, 2016; Chryst et al., 2018). However, an abundance of research in cognitive psychology tells us that attentional and memory systems can be biased, making retrospective self-reports prone to error (Raphael, 1987; Tourangeau, 1999). For example, positive events are remembered better than negative ones, highly emotional memories are easier to retrieve, and memories are even distorted by current motivations or context (Dutta and Kanungo, 2013; Matlin, 2016; Schacter et al., 2011). This recall bias can contribute to the incongruence between self-reported and objective measurements of environmental behavior (Kormos and Gifford, 2014). For example, farmers have been observed to exhibit recall bias in their environmental interactions and agricultural practices, resulting in self-reports that do not align with their actual actions (Beegle et al., 2011; Bell et al., 2019; Wollburg et al., 2021). To reduce recall bias, the time between the event of interest and self-report must be minimized. Additionally, environmental events may change dynamically, and if they are not sampled frequently enough, critical experiential and behavioral processes can be missed (Zhang et al., 2023). For instance, individuals' experiences with extreme weather events, such as wildfires or flooding, provide examples of the non-linear psychological processes that unfold over time.

Indeed, recent work has demonstrated that climate attitudes and behaviors can be influenced by recent or ongoing events such as perceived economic risk, perceived vulnerability to flooding, abnormal temperatures, and extreme weather events (Brooks et al., 2014; Hennes et al., 2016; Konisky et al., 2015; Spence et al., 2011). This demonstrates that contextual information, often not captured by traditional self-report measures, is critical to appreciate the underlying contributors of different climate attitudes and behaviors. In addition to context, changes over time are not captured by traditional single self-report measures. Climate change-related emotions have proven essential to shaping perception, motivating climate action, and influencing wellbeing (Böhm et al., 2023; Ogunbode et al., 2021; Reyes et al., 2021; Wong-Parodi and Feygina, 2021; Schneider and van der Linden, 2023) but are fleeting and can change dynamically within minutes to an hour (Colombo et al., 2020; Verduyn, 2021; Verduyn and Lavrijsen, 2015). Therefore, for a holistic understanding, it is critical to measure how affect, behaviors, and attitudes fluctuate and interact in a given environmental context. Yet, given the challenges of real-time longitudinal assessments, very limited research can capture dynamic changes in experiences, cognition, and behavior in naturalistic environmental contexts. Thus, extending previous calls for improved ecological validity (Burgess et al., 2006; Koehler, 1996; Parsons, 2016), we advocate for the adoption of EMA to capture contextually rich and frequent real-time assessments that will advance environmental science research.

### 1.3 An overview of ecological momentary assessment methodology

EMA is a methodology used in behavioral and affective science, often in a health context, because of the strengths it offers over other sampling techniques (e.g., Moskowitz and Young, 2006; Myin-Germeys et al., 2018; Palmier-Claus et al., 2019; Shiffman, 2007; Stone and Shiffman, 1994; Stone et al., 2023a). EMA typically involves repeated measurements to record multiple instances of the construct of interest, providing near real-time assessment of experiences and behaviors. EMA is an umbrella term that covers several mediums of data collection and timing, which are carefully selected to address the research question. For example, diaries, surveys, behavioral observations, random or scheduled experience sampling, or physiological monitors can all be utilized in EMA (Hand and Perzynski, 2016). The type of EMA data collected depends on the question of interest (Burke et al., 2017; Wrzus and Neubauer, 2023). Given the focus of environmental science research, it may sometimes be useful to collect data when specific events occur (e.g., instances when extreme weather events such as wildfires or flooding occur; i.e., event-contingent). A common approach is to prompt participants to respond to questions that are collected randomly (e.g., experiences sampled three times a day when beeped; i.e., signal-contingent). The frequency with which such measurements are collected varies greatly depending on the research question (e.g., several times a day or week). Contextual (e.g., cultural, social, physical, economic factors) or supplementary data (e.g., confounding factors that could potentially influence main variables of interest) can be collected throughout the study period for a more holistic understanding.

In terms of equipment, technological advances have made smartphones easily adaptable for EMA data collection (Yang et al., 2019). Smartphones can also be configured to collect additional information throughout the day, such as participants' location or health data, if paired with wearable health monitors (Lohani et al., 2025). Using participants' personal smartphones can allow researchers to make it convenient for respondents to participate in research, leading to lower missing data as they feel more user-friendly, and they can be set up with reminders for participants (Doherty et al., 2020). This also enables access to larger samples and reduces research costs (De Vries et al., 2021). However, researchers should consider providing equipment to those without access to smartphones to ensure equitable inclusion. There are costs and benefits to the different ways of conducting EMA, but they all aim to address some of the limitations associated with traditional self-report methodologies.

### 1.4 Benefits of adopting ecological momentary assessment: capturing intentions, actions, and context

One critical benefit of EMA is that it reduces memory and attentional biases by taking multiple measurements throughout the environmental event of interest. Recall bias is greatly reduced by minimizing the gap between the occurrence of events and assessment (Napa Scollon et al., 2009). Respondents are also prompted to evaluate their behaviors, feelings, or attitudes within the same context in which they occurred, reducing the influence of new and unrelated events. For example, EMA might capture a participant's feelings, stressors, and efforts toward conserving water during a drought rather than relying on their memory once the drought period is over. Additionally, desirability bias, or the tendency to respond according to social expectations, can lead participants to overestimate their adherence to environmental behaviors (ElHaffar et al., 2020). EMA allows participants to report their genuine thoughts and behaviors without the added influence of perceived social pressures, which can be triggered by direct interviews or the presence of researchers. Without social presence, biased responses due to social desirability are expected to be lower (Hand and Perzynski, 2016). EMA can also be set up to contact participants at times that work best for them, reducing survey fatigue and making data collection easier than traditional methods (Jones and Ballon, 2020). However, other researchers suggest that collected data at fixed times across participants may be a better choice as it reduces unsystematic variability across participant responses (Von Engelhardt and Jones, 2019). One suggested approach is to gain an understanding of availability from the target population and make decisions about EMA events collected to make comparison across participants feasible (Von Engelhardt and Jones, 2019). At the same time, this decision depends on the research question and the difficulty in collecting data. The researchers should make informed decisions and be transparent about the potential limitations of the choices made in their project.

Measuring intentions to engage in pro-environmental behavior is a good first step toward understanding when and why people support climate action. However, pro-environmental intentions alone are insufficient to mitigate the climate crisis, and concrete action is needed to bring about real change. Research on the environmental intention-behavior gap demonstrates that although there is a moderate to strong link between environmental intentions and actions (Bamberg and Möser, 2007; Klöckner, 2013), a significant gap still remains. Many people do not engage in environmental behavior at the rate that their intentions might suggest (ElHaffar et al., 2020; Frank and Brock, 2018). For example, intentions to shop sustainably do not always result in avoidance of environmentally harmful products (Park and Lin, 2020; Vermeir and Verbeke, 2006). Similarly, this discrepancy between intentions and actions has been observed in recycling, water conservation, and the adoption of renewable energy systems (Claudy et al., 2013; Dolnicar and Hurlimann, 2010; Echegaray and Hansstein, 2017). EMA can be used to help us understand both intentions and actions, including the differences between the two, by prompting participants to reflect on their actual behaviors multiple times throughout the study. Instead of relying on the assumption that people who intend to behave environmentally actually do behave environmentally, EMA allows us to view these constructs as related but separate. EMA methodology is similarly applicable to other environmental science domains more broadly. For example, research designs can be easily extended to other environmental behaviors, such as investigating carbon footprints, food waste, or recycling behaviors (Broers et al., 2021).

EMA can also capture various contextual factors that may differentially influence attitudes, intentions, and actions. Individuals might not consistently engage in environmentally responsible behavior across different contexts due to varying constraints, a sense of disconnect between behaviors, differing perceived impact on the climate, or the extent of personal costs involved (Thøgersen, 2004; Tobler et al., 2012; Whitmarsh et al., 2018). For instance, someone may recycle at home but not in their workplace due to a lack of recycling bins or a culture that discourages taking the time to separate trash from recycling. Within-subject changes and variability over time are not captured by the between-subject effects measured by traditional self-report methods; however, they can provide novel and valuable insights (Conner et al., 2009). Therefore, EMA enables researchers to track how people's responses change across contexts by gathering information from multiple personally relevant and naturalistic settings.

## 2 Discussion

### 2.1 Integration of ecological momentary assessment: how it can advance environmental science research

A few studies have already shown how EMA methodology can be successfully integrated into environmental science research. In the context of pro-environmental behaviors, researchers have shown how EMA can uncover novel findings that traditional self-report methods might easily overlook (Bissing-Olson et al., 2016; Tao et al., 2021). For example, Bissing-Olson et al. (2016) used EMA to uncover a relationship between pride and pro-environmental behavior. Perceived descriptive norms about pro-environmental behavior moderated this relationship, an association that would have likely been overlooked without consideration of context as allowed by EMA methodology.

Another study (Tao et al., 2021) employed EMA to evaluate air pollution exposure and stress levels among residents living in different types of housing in Beijing, China. Through the use of EMA methodology, this study captured daily variations in air pollution exposure and stress levels, leading to the conclusion that the impact of air pollution is not uniform across housing groups and is driven by differences in mobility and coping capacity. Without the use of EMA methodology, state residential assessments had in the past been unable to account for these daily variations and missed this finding completely.

The utility of EMA in accounting for fluctuations over time was also critical to the research conducted by Jones and Ballon (2020), which investigated the effects of flooding on different socio-economic groups in Myanmar. By carrying out successive phone surveys every 6 to 8 weeks for a period of 12 months, researchers were able to track rapid evolutions in resilience and recovery, including subtle changes from month to month that a normal survey would miss. In another recent work, experience sampling was successfully used to examine how rising temperatures were linked to mood changes (Bundo et al., 2023). Similarly, eco-emotions have been recently examined in everyday life in response to climate change (Contreras et al., 2024; Lohani et al., in press^1^; Lutz et al., 2023; Mathers-Jones and Todd, 2023; Meidenbauer et al., 2024). Studies such as these highlight the influential role that EMA can play in bolstering conclusions with critical environmental and health implications.

### 2.2 Illustrative examples that incorporate ecological momentary assessment in climate change research

To further illustrate how EMA can be adopted in environmental science research, we present several recent examples and potential extensions. In an effort to separate how environmental stressors may connect with daily wellbeing, a recent study adopted EMA to record how participants experienced daily stressors (Lohani et al., under review a^2^). When randomly prompted once every hour of a day, participants used their phones to report if they experienced any environmental stressors. This design facilitated a thorough evaluation of environmental stressors as they manifest in daily life and their possible effects on mental health. The findings suggested that experiences of environmental stressors were closely linked to lower everyday wellbeing. This study can further be extended to learn about additional predictors and covariates of interest. For example, participants could be asked to report different kinds of specific environmental stressful events (e.g., poor air quality, water contamination, irregular weather patterns, etc.) on good vs. bad air quality days, helping understand the direct links between environmental stressors and wellbeing. Relatedly, a recent study examined how global warming and irregular temperatures were linked to mood disorders (Clery et al., 2024). Additionally, participants' phones could be set up to collect sensor data (e.g., air quality or temperature; Tao et al., 2021), which can then be linked to associations with psychological responses such as climate distress. Participants could also have the option to report their experiences and behaviors when particular environmental events occurred. This would help better capture significant occurrences from the public's perspective.

In another application of EMA methodology, it can be adapted to investigate learning processes and outcomes around environmental science. For instance, in a recent study, university students' psychological responses to a lecture on scientific facts about climate change (Lohani et al., under review b^3^). This study helped understand the emotions and behaviors of students learning about the climate change crisis. Similarly, utilizing a more informal learning environment of a natural history museum, the psychological responses of museum visitors were gathered multiple times within an exhibit on climate change (Lohani et al., revise and resubmit). Future extensions of these studies could include follow-up assessments after students and museum visitors learn about the urgency of addressing climate change challenges. These follow-up EMAs could be used to capture any changes in attitudes, beliefs, and behaviors after participants learn about the climate change trajectory and its impact on their local community.

Overall, EMA incorporates studies that enable researchers to minimize memory and attentional bias (Kormos and Gifford, 2014) and learn about individual reactions, opinions, beliefs, and engagement in environmental science content in both formal and informal real-world settings. Additionally, they provide near-real-time information on psychological experiences and behavior around environmental events over the short- and long-term to gain a dynamic understanding. Together, these dynamic and personally relevant details can assist researchers in developing a more nuanced and contextually informed understanding of the relationship between affect, cognition, and behavior, which are interconnected within an environmental science matter.

### 2.3 A few limitations and considerations in adopting ecological momentary assessment into environmental science

So far, we have highlighted numerous benefits of EMA, but it would be remiss to overlook the limitations and challenges involved in adopting it for environmental research. When implementing EMA in environmental science research, several aspects need to be considered. First, it is vital to be mindful of the potential vulnerability of potential respondents and their reluctance to trust and share their experiences. Working with community partners to gain an understanding of any sensitive issues and ongoing challenges can help create a supportive environment. Coordinating data collection with the help of community partners can help maintain integral relationships with community members (Stonewall et al., 2020). Recruiting participants in conjunction with community organizations or in person may help increase trust and improve participation (Stonewall et al., 2020). Additional educational resources and opportunities are a fulfilling way to support community relationships and outreach.

Second, it is extremely critical to consider ethical concerns about collecting data from participants who are experiencing extremely challenging times in high-risk conditions. Lessons can be learned from past research ethics around humanitarian disasters, especially involving marginalized populations (e.g., Fisher, 2022; Mezinska et al., 2016; Mueller et al., 2023; O'Mathúna, 2010; Stonewall et al., 2020; Voss, 2008). Scholars have suggested that researchers should consider all opportunities to help distressed individuals (Dabalen et al., 2016; Jones and Ballon, 2020). Researchers should seek to gather valuable information and resources for participants in hazardous conditions and ensure that these sources are regularly shared. For example, the messaging system utilized by the researchers for EMA could also be used to provide helpful and urgent information during times of crisis. Similarly, the participants could use the EMA application to communicate with others and exchange useful information. Data collection for a study should be postponed if there are any safety concerns due to a major environmental crisis or event.

Third, several logistical factors must be considered in advance before data collection is initiated. The medium of data collection would depend on the availability of Wi-Fi and electronic devices (e.g., phones), and researchers will need to adapt to a suitable format to accommodate any environmental restrictions (e.g., due to extreme weather events such as wildfires or flooding). Sometimes, when there is no power or Wi-Fi, researchers may need to collect data on paper or use daily diary approaches. If logistical conditions allow, participants could be given a phone or EMA device that may help overcome exclusions due to socioeconomic limitations.

At the same time, it is worth considering the time limitations and emotional toll individuals (especially those in vulnerable situations) may face if EMAs probe sensitive topics. Similar to research involving challenging events or sensitive issues, careful consideration of the information obtained and the potential harm caused by the EMA items is essential. Providing participants with relevant explanations for the rationale of the study and its potential to advance science could be provided if applicable. It can also be tricky to decide on the fitting compensation provided to reimburse participants for their time, effort, and any inconvenience incurred. However, factors such as the duration and complexity of the study should certainly be considered.

Fourth, the EMA methodology effectively provides a detailed perspective on changes in constructs over time; however, it is important to consider whether the frequent assessment itself could potentially influence the construct of interest. For example, it is possible that some changes in emotions over the course of the day may be due to the frequent assessments themselves. Relatedly, EMA has been shown to reduce memory and attentional biases, as well as social desirability, but it is important to be mindful that such biases are not completely eliminated. These issues remain a challenge for any kind of psychological assessment methodology.

Fifth, it is useful to balance the importance of a number of variables, the length of questions, and the frequency of assessment being asked that can contribute to response burden (Smyth et al., 2021; Stone et al., 2023b; Yan et al., 2019). If assessments are too long or frequent, the respondents may become less compliant over time and even stop responding (Tate et al., 2024; McCarty et al., 2006). Hence, more measurements (without theoretical rationale) are not necessarily better and may lead to higher rates of missing data and dropout. Given the repeated nature of data collection, it is possible that participants may respond to the same event more than once (especially if they are too close in time). Anticipating this issue is important when determining the frequency of assessment that is most meaningful for the research questions. Similarly, clear instructions on what is being asked can help prevent messy data. Whenever possible, it is best to ensure that the respondents understand what is being asked of them. There should also be a mechanism to ask follow-up questions (e.g., phone contact information), as some participants may need additional support over time.

Sixth, both quantitative and qualitative data can be successfully collected using the EMA methodology. In addition to the already requested structured questions, it is helpful to provide respondents with the option to document qualitative messages when they wish to report qualitative data. This can allow researchers to capture novel details as they occur over time in respondents' naturally occurring settings. Moreover, EMA technology can be combined with environmental sensors (e.g., temperature and air quality) to help implement innovative ways of integrating environmental and psychological variables together (e.g., Wilhelm and Grossman, 2010; Hoemann et al., 2020). Indeed, in past work, participants were given mobile phones with GPS and ambulatory air sensors to capture PM2.5 concentrations over time (Tao et al., 2021). Taking this study as an example, future work could complement air quality and location data streams by collecting EMA data to understand air quality-related perceptions, cognitions, and actions simultaneously. Collectively, such a multi-method approach can provide a comprehensive understanding of the underlying processes critical to advancing environmental science.

### 2.4 Concluding remarks

This paper presents literature demonstrating that EMA provides an ecological approach to capturing how humans experience and behave in their naturally occurring environment over time. The EMA methodology is particularly beneficial for understanding environmental variables that change quite dynamically and are prone to getting convoluted or lost otherwise, such as fluctuating environmental conditions. Another key advantage is the feasibility of collecting reliable data in the real world, which drastically improves the ecological relevance of findings. Furthermore, EMA allows measurements across multiple environmental events and helps to connect intentions and feelings to actions. Drawing from climate science, the paper suggests that integrating the EMA methodology into environmental science research will generate new knowledge about the challenges of climate change and the effectiveness of climate adaptation and mitigation efforts. In sum, EMA is an innovative methodology for advancing interdisciplinary environmental science research.

## Data Availability

The original contributions presented in the study are included in the article/supplementary material, further inquiries can be directed to the corresponding author.

## Footnotes

^1^Lohani, M., Zummo, L., Janney, B. A., and Giron, J. (in press). Exploring emotional reactions and regulation strategies in climate change contexts: insights from a museum exhibit. *J. Museum Educ*.

^2^Lohani, M., Elsey, J., Dutton, S., and Zummo, L. (under review a). Climate change is linked to daily wellbeing: The role of environmental, governmental, and commute-related stressors.

^3^.Lohani, M., Zummo, L., Brunelle, A., Banerjee, D., Cachelin, A., Yeo, S., and Shah, J.F. (under review b). Together, we learn and make a difference: Emotion regulation strategies among climate science students.
